# Cannabis Use Induces Distinctive Proteomic Alterations in Olfactory Neuroepithelial Cells of Schizophrenia Patients

**DOI:** 10.3390/jpm11030160

**Published:** 2021-02-25

**Authors:** Marta Barrera-Conde, Karina Ausin, Mercedes Lachén-Montes, Joaquín Fernández-Irigoyen, Liliana Galindo, Aida Cuenca-Royo, Cristina Fernández-Avilés, Víctor Pérez, Rafael de la Torre, Enrique Santamaría, Patricia Robledo

**Affiliations:** 1 Integrative Pharmacology and Systems Neuroscience, Neuroscience Research Program, IMIM-Hospital del Mar Research Institute, 08003 Barcelona, Spain; mbarrera1@imim.es (M.B.-C.); acuenca@imim.es (A.C.-R.); RTorre@imim.es (R.d.l.T.); 2 Department of Experimental and Health Sciences, University Pompeu Fabra, 08003 Barcelona, Spain; cristina.fernandeza@upf.edu; 3 Clinical Neuroproteomics Unit, Proteomics Platform, Navarrabiomed, Complejo Hospitalario de Navarra (CHN), Universidad Pública de Navarra (UPNA), IdisNA, Proteored-ISCIII, 31006 Pamplona, Spain; karina.ausin.perez@navarra.es (K.A.); mercedes.lachen.montes@navarra.es (M.L.-M.); jokfer@gmail.com (J.F.-I.); enrique.santamaria.martinez@navarra.es (E.S.); 4 Department of Psychiatry, University of Cambridge, Cambridgeshire and Peterborough NHS Foundation Trust, Cambridge CB2 1TN, UK; lg532@cam.ac.uk; 5 Neuropsychiatry and Addictions Institute (INAD) of Parc de Salut Mar, 08003 Barcelona and CIBER de Salud Mental, Spain; 61155@parcdesalutmar.cat; 6 Department of Psychiatry and Legal Medicine, Autonomous University of Barcelona, 08193 Barcelona, Spain; 7 Centro de Investigación Biomédica en Red de Fisiopatología de la Obesidad y Nutrición, Instituto de Salud Carlos III, 28029 Madrid, Spain

**Keywords:** cannabis, schizophrenia, proteomics, olfactory neuroepithelium, metabolism, RNA, ZNF326, MTREX

## Abstract

A close epidemiological link has been reported between cannabis use and schizophrenia (SCZ). However, biochemical markers in living humans related to the impact of cannabis in this disease are still missing. Olfactory neuroepithelium (ON) cells express neural features and offer a unique advantage to study biomarkers of psychiatric diseases. The aim of our study was to find exclusively deregulated proteins in ON cells of SCZ patients with and without a history of cannabis use. Thus, we compared the proteomic profiles of SCZ non-cannabis users (SCZ/nc) and SCZ cannabis users (SCZ/c) with control subjects non-cannabis users (C/nc) and control cannabis users (C/c). The results revealed that the main cascades affected in SCZ/nc were cell cycle, DNA replication, signal transduction and protein localization. Conversely, cannabis use in SCZ patients induced specific alterations in metabolism of RNA and metabolism of proteins. The levels of targeted proteins in each population were then correlated with cognitive performance and clinical scores. In SCZ/c, the expression levels of 2 proteins involved in the metabolism of RNA (MTREX and ZNF326) correlated with several cognitive markers and clinical signs. Moreover, use duration of cannabis negatively correlated with ZNF326 expression. These findings indicate that RNA-related proteins might be relevant to understand the influence of cannabis use on SCZ.

## 1. Introduction

The complexity of schizophrenia (SCZ), involving intricate interactions between environmental factors and genetics, hinders the identification of the molecular mechanisms underlying its development [1]. State of the art techniques are being used to find biomarkers and ease clinical practice. In this sense, proteomic tools, allowing the study of a large number of proteins at a time, can provide an integrated picture of the biological dysfunctions associated with SCZ [2]. Serum, plasma and cerebrospinal fluid studies reveal an effect on proteins related to the innate immune system and a highly reported downregulation of Apolipoprotein A-I1 (APOA1) [3,4,5], which might mediate the impact of SCZ on the peripheral immune response [6,7]. On the other hand, postmortem brain studies have shown deregulations in metabolic pathways, calcium signalling and cytoskeleton assembly in several brain areas in SCZ [8,9,10]. However, other neuropsychiatric conditions present similar affectations [11]. In fact, the two most referenced biomarkers of SCZ, namely Fructose-bisphosphate aldolase C (ALDOC) and Glial fibrillary acidic protein (GFAP), are also altered in major depression and bipolar disorder [12]. Thus, postmortem brains from individuals diagnosed with different mental conditions appear to have great similarities that hamper the identification of exclusive biomarkers for SCZ. Additionally, the impossibility of correlating these biochemical findings with cognitive dysfunctions and environmental cues specific for each condition highlights the necessity to use other substrates. In this context, the olfactory neuroepithelium (ON) has emerged as a useful tool. This specialized epithelial tissue can be easily obtained from living subjects and it contains multipotent progenitors that express neural markers [13,14,15,16]. Therefore, the main advantage of ON cells is that ongoing environmental factors and molecular alterations can be evaluated during the progression of the disease. 

Years of epidemiological research in psychotic disorders suggest that cannabis use is a specific environmental factor for SCZ. The incidence of cannabis use is two to four times higher among SCZ patients, and its consumption doubles the risk of psychosis in a dose- and time-dependent manner [17,18]. Moreover, an endocannabinoid system imbalance, including alterations in the expression of the cannabinoid receptor 1 (CB_1_) in postmortem brains and a diminished production of the enzymes involved in the synthesis of endocannabinoids, has been observed in postmortem and peripheral samples of SCZ patients [19,20]. Additionally, cannabis and SCZ have a shared effect on some cellular functions. Indeed, protein metabolism via AKT/mTOR, cytoskeleton organization, and calcium signalling are affected in human samples from SCZ patients [2], and after cannabinoid exposure in cultured cells [21,22] and mice models [23,24]. Moreover, plasmatic samples from heavy cannabis users and SCZ patients share an effect on oxidative stress response [7,25]. However, whether the coexistence of both factors has equal or distinct consequences is less clear. In fact, in a synchrotron-based infrared spectroscopy study, significant differences in the protein spectra were reported in ON cells from SCZ cannabis users compared to non-users [26]. Additionally, we recently demonstrated that the functional signature of the CB_1_ and serotonin 2A receptor (CB_1_-5HT_2A_R) heteromer in ON cells is differentially regulated in SCZ patients depending on cannabis use [27]. 

Therefore, the aim of this study was to find exclusively deregulated proteins in ON cells of SCZ patients with and without a history of chronic cannabis use. We applied mass spectrometry-based quantitative proteomics followed by a functional analysis to characterize the proteomic changes specifically induced by cannabis use in ON cells from SCZ patients, as compared to controls that use cannabis or not. The primary outcome of the study, i.e., the expression levels of the target proteins, were correlated with cannabis consumption, and with secondary cognitive and clinical outcomes associated with SCZ. 

## 2. Materials and Methods

### 2.1. Study Design

SCZ patients without a history of chronic cannabis use (SCZ/nc, *n* = 5), SCZ patients with a reported history of chronic cannabis use (SCZ/c, *n* = 5), chronic cannabis users without any psychiatric diagnosis (C/c, *n* = 5), and control subjects non-cannabis users (C/nc, *n* = 5) between 18 and 45 years old (both males and females), were recruited to conduct a cross-sectional study. Every subject gave written informed consent after a complete description of the study and the procedures involved. Cannabis users had to consume more than 5 cannabis cigarettes per week during at least 6 months. On the day of testing, subjects were told to refrain from cannabis use for at least 12 h before testing to avoid the potential confounding of acute cannabis intoxication in both neuropsychological and proteomic studies. The exclusion criteria were: (i) meeting criteria for any severe mental disorder according to the Diagnostic and Statistical Manual of Mental Disorders, Fifth Edition (DSM-5); (ii) history of severe mental illness among first degree relatives; (iii) history of severe congenital, medical or neurological illness; (iv) show medical conditions with nasal repercussions (rhinitis or bleeding); and (v) use of other drugs of abuse. SCZ-diagnosed participants were receiving antipsychotic medication at the time of the study. All subjects underwent a physical, psychiatric and neuropsychological evaluation, as previously described [16]. This study was approved by the local Institutional ethics committee (CEIC-PSMAR).

### 2.2. Clinical and Neuropsychological Evaluation

Neuropsychological assessments were carried out in all subjects. Executive functions were tested with the semantic verbal fluency test. Attention performance was evaluated using the direct spatial span (SSP) and digit direct series. Working memory was addressed using the inverse spatial span and digit inverse series using the Cambridge neuropsychological test automated battery (CANTAB 2017), and the digit span of the Wechsler Adult Intelligence Scale (WAIS-III). Emotional recognition was evaluated with the CANTAB test. Clinical outcomes included the global assessment of functioning (GAF), and the neurological soft signs (NSS) scores.

### 2.3. Quantification of Cannabis Metabolites

To estimate the amount of cannabis consumed by cannabis users, the plasma concentrations of its main non-psychoactive metabolite, namely, 11-nor-9-carboxy-Δ-9-THC (THC-COOH), were calculated. Briefly, 1 mL of plasma was transferred into a glass tube and spiked with d3-Δ-9-THC as the internal standard. A protein precipitation with 2 mL of 0.1% formic acid in acetonitrile was performed prior to a solid phase extraction with Oasis Prime HLB 3 cm^3^, 60 mg column (Waters Co., Milford, MA, USA). The supernatant was diluted with MilliQ water and loaded. Subsequently, 2 mL of 25% of methanol was added twice. Elution was carried out twice with 2 mL of 90:10 acetonitrile:methanol (ACN:MeOH). The organic phase was evaporated to dryness under a nitrogen stream at < 39 °C and <15 psi pressure. Analytes were reconstituted in 50 μL of 90:10 ACN:MeOH and 50 μL of MilliQ water. Quantification of THC-COOH in plasma was performed using an Agilent 1200 series HPLC system (Agilent Technologies) coupled to a 6410 Triple Quadrupole LC-MS (Agilent Technologies) mass spectrometer with an electrospray interface. 

### 2.4. Nasal Exfoliation and Cell Culture

ON samples were obtained by nasal brushing. Samples from the middle and upper turbinates were maintained in 250 µL of cold Dulbecco’s Modified Eagle Medium/Ham F-12 (DMEM/F12) enriched with 10% FBS, 2% glutamine and 1% streptomycin–penicillin (GibcoBRL), as previously described [27]. At 80% confluence, cells were expanded using 0.25% trypsin (GibcoBRL) and replated in 75 cm^2^ flasks. Then, cells were expanded until passage four was reached and stored in liquid nitrogen with 20% FBS and 10% dimethyl sulfoxide (Sigma-Aldrich, Madrid, Spain). The aliquots from all subjects (*n* = 20) were unfrozen at the same time and harvested in 75 cm^2^ flasks under standard conditions using enriched DMEM/F12 medium. 

### 2.5. Quantitative Proteomics in ON Cells

Physical protein extraction was performed using sterile scrappers and PBS 1X. After centrifugation, the pellet was kept frozen at −80 °C until homogenization using lysis buffer (7 M urea, 2 M thiourea, and 50 mM DTT). The homogenates were then subjected to in-solution digestion, peptide purification and reconstitution prior to mass spectrometric analysis (MS/MS). Data acquisition was performed as previously described [28] using ProteinPilot v5.0 (Sciex) as a search engine, ParagonTM Algorithm (v.4.0.0.0) [29] for database searching and a non-lineal fitting method to calculate the false discovery rate (FDR) (1% Global FDR or better). Data analysis was performed by sequential window acquisition of all theoretical mass spectra–mass spectrometry (SWATH-MS), and the TripleTOF 5600+ mass-spectrometer was configured as previously described [30]. The library generation-associated ProteinPilot group file was loaded into PeakView^®^ 2.1 (Sciex), and peaks from SWATH runs were extracted with a peptide confidence threshold of 99% confidence (Unused Score ≥1.3) and FDR lower than 1%. ProteinPilot was used to extract the MS/MS spectra of the assigned peptides, and only proteins quantified with at least two unique peptides were considered. For more detailed information about the SWATH-MS library generation, please refer to the extended experimental procedures in the Supplementary Information.

### 2.6. Pathway Analysis, Statistics and Bioinformatics

The peer-reviewed pathway database REACTOME [31] was used to functionally characterize the proteomic alterations detected in every comparison. This database hierarchically classifies cellular functions in 27 cascades. We established a 0.01 *p*-value threshold, and the functional proteome results obtained were ordered based on their FDR. All calculations were performed using SPSS (SPSS Inc., Chicago, IL, USA). Normality and homoscedasticity for continuous variables were tested using Shapiro–Wilk W and Levene tests. Demographic categorical variables were evaluated using Chi-squared tests and continuous normally distributed variables were evaluated using one-way ANOVAs followed by the least significance difference (LSD) post-hoc test. Neuropsychological calculations were corrected for tobacco use duration. Not normally distributed variables were compared using Kruskal–Wallis and Dunn’s post-hoc test. Vulcano plot representations and Spearman correlation plots were depicted using GraphPad prism 8.0 Software (La Jolla California, CA, USA). Venn diagrams for proteomic comparisons were designed using BioVenn platform [32].

## 3. Results

### 3.1. Demographics and Neuropsychological Outcomes

The demographic data are shown in Table 1. The groups did not differ in age, sex or dosage of tobacco use. However, SCZ/c showed a significantly higher duration of tobacco use (years) when compared to all other groups (*p* < 0.05). The analysis of cannabis use patterns between C/c and SCZ/c revealed no significant differences. The neuropsychological evaluation (Table 2) revealed significantly lower verbal fluency scores for SCZ/nc and SCZ/c as compared to C/nc (*p* < 0.05), but not when compared to C/c. GAF scores were significantly lower in SCZ/nc and SCZ/c when compared to C/nc and C/c (*p* < 0.001). Finally, significantly more NSS were present in SCZ patients regardless of cannabis use when compared to C/nc (*p* < 0.01) and C/c (*p* < 0.05). 

### 3.2. Plasmatic Concentrations of THC-COOH

The plasmatic concentrations of THC-COOH were not significantly different between the groups (C/c: 34.92 ± 17.55 ng/mL; SCZ/c: 11.22 ± 15.91 ng/mL).

### 3.3. Proteomic Analyses

Our proteomic workflow was fundamentally based on a triangular approach [33], whereby a relatively small number of cases and controls are analyzed by hypothesis-free discovery proteomics in great depth, leading to the quantification of thousands of proteins. Using this workflow, we observed specific changes in protein abundance in C/nc vs. SCZ patients and in SCZ/nc vs. SCZ/c, underpinning the importance of analyzing independent factors (disease/cannabis), instead of merely contrasting SCZ versus control cases. Firstly, C/c, SCZ/nc and SCZ/c were compared to C/nc subtracting exclusively deregulated proteins for each population. Then, each group of SCZ patients was compared to C/c to subtract the effect of cannabis use on control subjects. In the end, each group of SCZ (SCZ/nc and SCZ/c) was compared to obtain proteomic biomarkers that were only affected by SCZ or by SCZ plus cannabis use. For the summary followed in the proteomic data analysis, see Supplementary Figure S1. 

#### 3.3.1. Quantitative and Functional Proteomic Profile of ON Cells from SCZ Patients as Compared to C/nc

**Figure 1:**
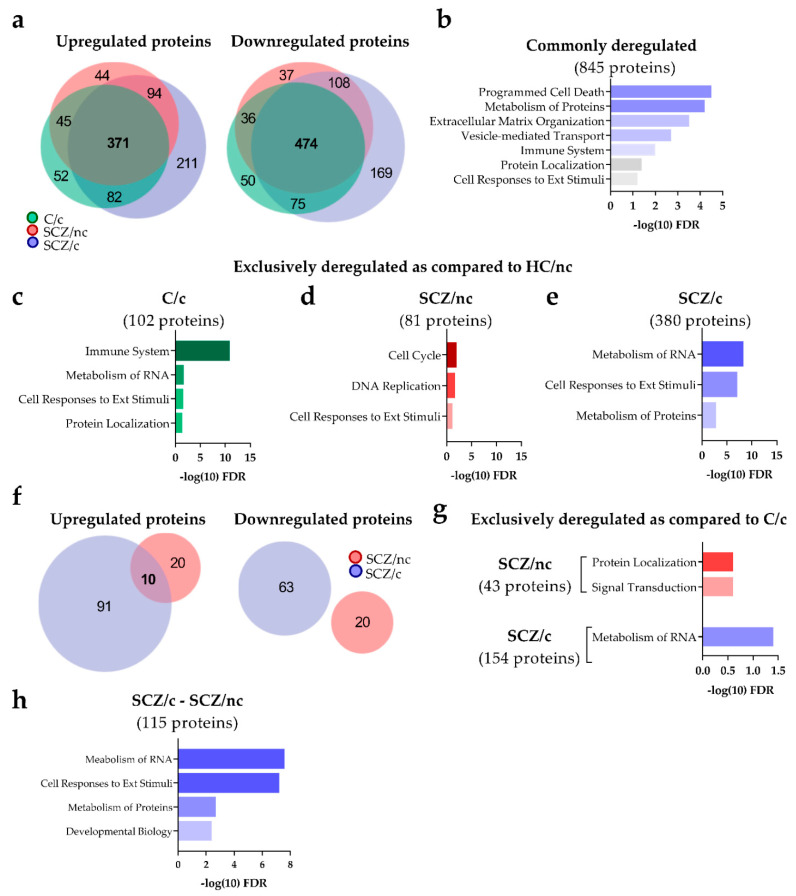
Characterization of the proteome of ON cells from control subjects non-cannabis users (C/nc), control cannabis users (C/c), schizophrenia patients non-cannabis users (SCZ/nc) and schizophrenia patients cannabis users (SCZ/c). (**a**) Proteomic alterations in C/c, SCZ/nc and SCZ/c as compared to C/nc. The upregulated and downregulated proteins in C/c-C/nc, SCZ/nc-C/nc and SCZ/c-C/nc are represented in two different Venn diagrams. (**b**) Enrichment pathway analysis of the 845 proteins commonly deregulated in C/c, SCZ/nc and SCZ/c as compared to C/nc. (**c**) Enrichment pathway analysis of the exclusively deregulated proteins in C/c, (**d**) SCZ/nc (**e**) and SCZ/c versus C/nc. (**f**) Proteomic alterations in SCZ/nc and SCZ/c as compared to C/c. The upregulated and downregulated proteins when SCZ/nc-C/c and SCZ/c-C/c are compared are represented in two Venn diagrams. (**g**) Functional characterization of the 43 proteins deregulated in SCZ/nc and the 154 proteins deregulated in SCZ/c as compared to C/c. (**h**) Functional characterization of the 115 proteins deregulated in SCZ/c as compared to SCZ/nc. Data from the pathway analysis are represented as minus the logarithm (10) of the FDR.

The proteomic analysis of ON cells from C/c, SCZ/nc and SCZ/c as compared to C/nc showed 1185, 1209 and 1584 deregulated proteins, respectively. The Venn diagrams (Figure 1a) showed that in C/c, 550 proteins were upregulated (554 in SCZ/nc and 758 in SCZ/c). On the other hand, 635 proteins were downregulated in C/c (655 in SCZ/nc and 826 in SCZ/c); 371 proteins were commonly upregulated in C/c, SCZ/nc and SCZ/c; 211 were exclusively upregulated in SCZ/c and 44 in SCZ/nc. In addition, 474 proteins were commonly downregulated in C/c, SCZ/nc and SCZ/c; 169 were exclusively downregulated in SCZ/c and 37 in SCZ/nc. In total, 845 proteins were commonly deregulated in C/c, SCZ/nc and SCZ/c as compared to C/nc. The pathway analysis of the 845 commonly deregulated proteins (Figure 1b) revealed programmed cell death (37 proteins; log_(10)_FDR = 4.5), metabolism of proteins (225 proteins; log_(10)_FDR = 4.2), extracellular matrix organization (47 proteins; log_(10)_FDR = 3.5), vesicle-mediated transport (87 proteins; log_(10)_FDR = 2.7), immune system (241 proteins; log_(10)_FDR = 2.0), protein localization (22 proteins; log_(10)_FDR = 1.4) and cellular responses to external stimuli (67 proteins; log_(10)_FDR = 1.2).

The exclusive impact of cannabis on C/c was represented by 102 proteins (Figure 1c), wherein the main pathways affected included the immune system (79 proteins; log_(10)_FDR = 10.9), metabolism of RNA (21 proteins; log_(10)_FDR = 1.7), cellular responses to external stimuli (19 proteins; log_(10)_FDR = 1.6) and protein localization (7 proteins; log_(10)_FDR = 1.4). When comparing the two groups of SCZ vs. C/nc, we detected a substantial difference (Figure 1d). SCZ/nc only showed 81 exclusively deregulated proteins, whereas 380 were deregulated in SCZ/c. The functional characterization of the 81 proteins deregulated in SCZ/nc indicated that most of them were involved in cell cycle (16 proteins; log_(10)_FDR = 2.0), DNA replication (6 proteins; log_(10)_FDR = 1.7) and cellular responses to external stimuli (11 proteins; log_(10)_FDR = 1.2). On the other hand, the 380 proteins deregulated in SCZ/c (Figure 1e) were associated with metabolism of RNA (61 proteins; log_(10)_FDR = 8.3), cellular responses to external stimuli (54 proteins; log_(10)_FDR = 7.1) and metabolism of proteins (104 proteins; log_(10)_FDR = 2.8).

#### 3.3.2. Quantitative and Functional Proteomic Profile of ON Cells from SCZ Patients as Compared to C/nc

To identify disease-exclusive protein markers, and tease out the effect of cannabis use, the exclusively deregulated proteins in SCZ/nc and SCZ/c were compared to C/c (Figure 1f). In total, 53 proteins were deregulated in SCZ/nc, whereas 164 proteins were differentially expressed in SCZ/c. In SCZ/nc, 30 proteins were upregulated and 101 in SCZ/c.

On the other hand, 23 proteins were downregulated in SCZ/nc and 63 in SCZ/c. The Venn diagram revealed that only 10 proteins were commonly upregulated in SCZ/nc and SCZ/c as compared to C/c, wherein none were commonly downregulated. The functional characterization of the 43 exclusively deregulated proteins in SCZ/nc (Figure 1g) indicated that signal transduction (2 proteins; log_(10)_FDR = 0.6) and protein localization (2 proteins; log_(10)_FDR = 0.6) were the mainly enriched functions. The 154 exclusively deregulated proteins in SCZ/c unveiled metabolism of RNA (21 proteins; log_(10)_FDR = 1.4) as the only deregulated cascade. Thus, the profile of deregulated protein pathways in SCZ/c patients as compared to C/c is quantitatively larger and functionally different to the profile observed in SCZ/nc. To further evaluate the specific proteins involved SCZ/nc vs. SCZ/c, we assessed their functional differences. The results showed that 115 proteins were differentially expressed (Figure 1h). These proteins were associated with metabolism of RNA (28 proteins; log_(10)_FDR = 7.6), cellular responses to external stimuli (27 proteins; log_(10)_FDR = 7.2), metabolism of proteins (40 proteins; log_(10)_FDR = 2.7) and developmental biology (25 proteins; log_(10)_FDR = 2.4). A detailed list of the proteins involved in each comparison can be found in the Supplement Material.

#### 3.3.3. Specific Proteins Markers of SCZ Depending on Cannabis Use

**Figure 2:**
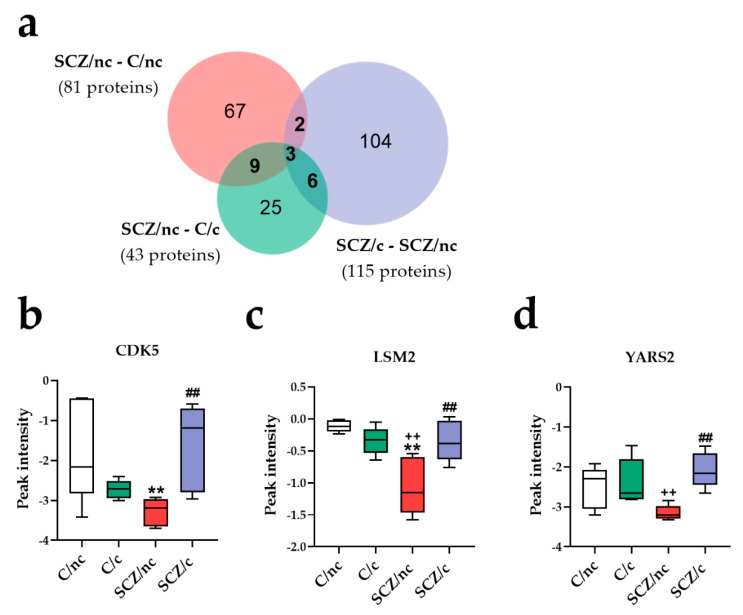
Specific proteomic markers of schizophrenia patients non-cannabis users (SCZ/nc). (**a**) Venn diagram comparing SCZ/nc to control subjects non-cannabis users (C/nc), control cannabis users (C/c) and schizophrenia patients cannabis users (SCZ/c). Graphical representation of CDK5 (**b**), LSM2 (**c**) and YARS2 (**d**), which were commonly deregulated in SCZ/c as compared to C/nc, C/c and SCZ/nc. ** *p* < 0.01; versus C/nc; ++ *p* < 0.01 versus C/c; ## *p* < 0.01 versus SCZ/nc.

To identify specific protein markers in ON cells of SCZ/nc, we designed a Venn diagram including the 81 proteins exclusively deregulated in SCZ/nc as compared to C/nc; the 115 proteins differently expressed in SCZ/nc as compared to SCZ/c; and the 43 proteins exclusively different between SCZ/nc and C/c (Figure 2a). In total, 3 proteins were commonly deregulated in these comparisons: (i) Cyclin-dependent-like kinase 5 (CDK5), (ii) LSM2 Homolog, U6 Small Nuclear RNA and mRNA Degradation-Associated (LSM2), and (iii) Tyrosine-tRNA ligase, mitochondrial (YARS2). The expression of CDK5 was significantly reduced in SCZ/nc (*p* < 0.01) as compared to C/nc. In addition, a significant increase in the expression of this protein was observed in SCZ/c when compared to SCZ/nc (*p* < 0.01) (Figure 2b). LSM2 was downregulated in SCZ/nc as compared to C/nc (*p* < 0.01) and vs. C/c (*p* < 0.01), and presented a different expression in SCZ/c vs. SCZ/nc (*p* < 0.01) (Figure 2c). YARS2 was significantly downregulated in SCZ/nc as compared to C/c (*p* < 0.01), and vs. SCZ/c (*p* < 0.01) (Figure 2d).

**Figure 3:**
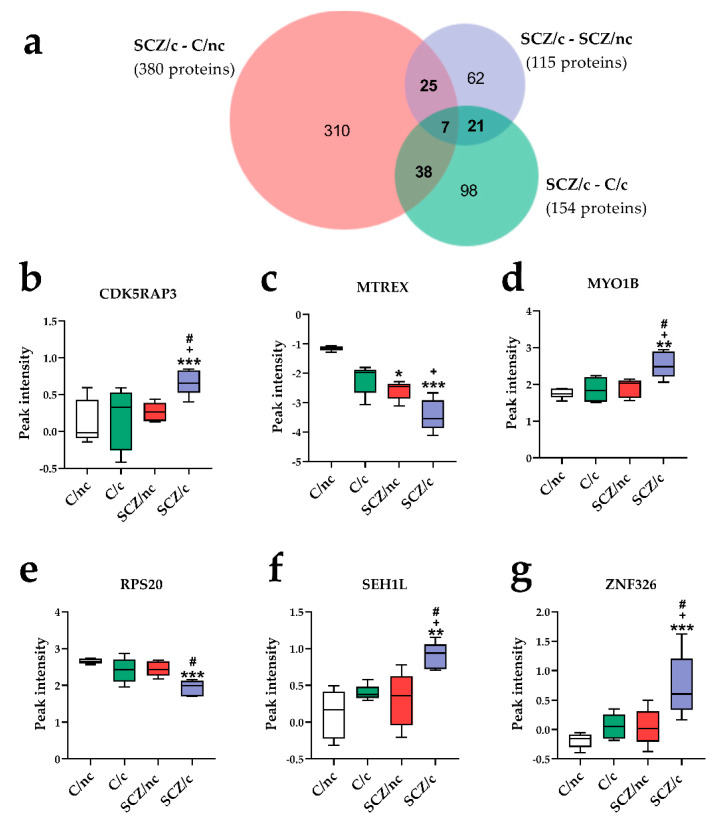
Specific proteome markers of schizophrenia patients cannabis users (SCZ/c). (**a**) Venn diagram comparing SCZ/c to control subjects non-cannabis users (C/nc), schizophrenia patients non-cannabis users (SCZ/nc), and control cannabis users (C/c). Graphical representation of CDK5RAP3 (**b**), MTREX (**c**), MYO1B (**d**), RPS20 (**e**), SEH1L (**f**), and ZNF326 (**g**), which were commonly deregulated in SCZ/c as compared to C/nc, C/c and SCZ/c. Data are represented as the mean ± minimum to maximum peak intensity. * *p* < 0.05; ** *p* < 0.01; *** *p* < 0001 versus C/nc; + *p* < 0.05 versus C/c; # *p* < 0.05; versus SCZ/nc.

To identify specific protein markers in the ON cells of SCZ/c, the 380 proteins exclusively deregulated in SCZ/c as compared to C/nc, the 115 proteins differently expressed in SCZ/c as compared to SCZ/nc, and the 154 proteins exclusively differing between SCZ/c and C/c, were compared (Figure 3a). Seven proteins were commonly deregulated in these three comparisons; (i) CDK5 regulatory subunit-associated protein 3 (CDKRAP3); (ii) Haloacid dehalogenase-like hydrolase domain-containing 5 (HDHD5); (iii) Exosome RNA helicase MTR4 (MTREX); (iv) Unconventional myosin-Ib (MYO1B); (v) 40S ribosomal protein S20 (RPS20); (vi) Nucleoporin SEH1 (SEH1L), and (vii) DBIRD complex subunit ZNF326 (ZNF326). CDK5RAP3 was significantly upregulated in SCZ/c as compared to C/nc, C/c and SCZ/nc (*p* < 0.001, *p* < 0.05, *p* < 0.05, respectively) (Figure 3b). HDHD5 was not significantly altered when the four groups were compared together. MTREX was downregulated in SCZ/c (*p* < 0.001) and SCZ/nc (*p* < 0.05) as compared to C/nc. SCZ/c showed a significant downregulation in its expression as compared to C/c (*p* < 0.05) (Figure 3c). MYO1B was significantly upregulated in SCZ/c as compared to C/nc (*p* < 0.01) and C/c and SCZ/nc (*p* < 0.05) (Figure 3d). RPS20 was downregulated in SCZ/c only, as compared to non-cannabis users (*p* < 0.001 vs. C/nc and *p* < 0.05 vs. SCZ/nc) (Figure 3e). SEH1L was significantly upregulated in SCZ/c as compared to C/nc (*p* < 0.01), C/c and SCZ/nc (*p* < 0.05) (Figure 3f). Finally, ZNF326 was significantly upregulated in SCZ/c as compared to C/nc (*p* < 0.001) and C/c and SCZ/nc (*p* < 0.05) (Figure 3g). 

### 3.4. Correlations between Specific Protein Markers, Cannabis Use and Cognitive Performance

**Figure 4:**
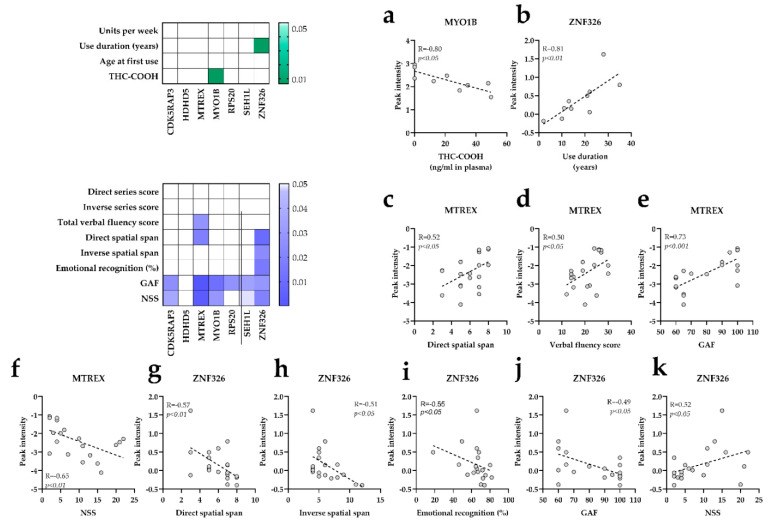
Association studies of protein markers, cannabis use and neurocognitive performance. (**a**) THC-COOH (ng/mL plasma) and MYO1B correlation. (**b**) Use duration and ZNF326 spearman correlation. (**c**) MTREX and direct spatial span correlation. (**d**) MTREX and verbal fluency score correlation. (**e**) MTREX and GAF correlation. (**f**) MTREX and NSS correlation. (**g**) ZNF326 and direct spatial span correlation. (**h**) ZNF326 and inverse spatial span correlation. (**i**) ZNF326 and emotional recognition correlation. (**j**) ZNF326 and GAF correlation. (**k**) ZNF326 and NSS correlation. In the correlation matrixes, only the significant correlations are colored. Darker squares indicate lower p-values.

A Spearman correlation plot was designed to evaluate the association between the expression levels of target proteins identified in SCZ/nc and in SCZ/c with cognitive performance, cannabis use patterns, and clinical signs in the entire population. For proteins related to SCZ/nc, the results showed that higher LSM2 expression was correlated with better GAF (r = 0.50, *p* < 0.05) and less NSS (r = −0.54, *p* < 0.05), whereas higher YARS2 levels were associated with better attentional performance in the direct series score test (r = 0.53, *p* < 0.05) (Data not shown). For proteins related to SCZ/c, MYO1B negatively correlated with plasmatic THC-COOH concentration (r = −0.80, *p* < 0.05) (Figure 4a), and ZNF326 positively correlated with cannabis use duration (r = 0.81, *p* < 0.01) (Figure 4b). MTREX showed a positive correlation with attentional performance in the direct spatial span test (r = 0.52, *p* < 0.05) (Figure 4c), and with better verbal fluency scores (r = 0.50, *p* < 0.05) (Figure 4d). Moreover, MTREX positively correlated with GAF (r = 0.73, *p* < 0.001) (Figure 4e), and correlated negatively with NSS (r = −0.65, *p* < 0.01) (Figure 4f). In addition, ZNF326 negatively correlated with attention in the direct spatial span test (r = −0.57, *p* < 0.01) (Figure 4j); with working memory in the inverse spatial span test (r = −0.51, *p* < 0.05) (Figure 4g); and with emotional recognition scores (r = −0.55, *p* < 0.05) (Figure 4h). Finally, higher ZNF326 correlated with lower GAF (r = −0.49, *p* < 0.05) (Figure 4i) and higher NSS (r = 0.52, *p* < 0.05) (Figure 4j). CDK5RAP3 correlated with GAF (r = −0.5, *p* < 0.05) and NSS (r = 0.47, *p* < 0.05) (Supplement Figure S2a,b). MYO1B levels were negatively correlated with GAF scores (r = −0.56, *p* < 0.05) and positively with NSS (r = 0.48, *p* < 0.05) (Supplementary Figure S2c,d). Finally, RPS20 was positively associated with GAF (r = −0.50, *p* < 0.05) (Supplementary Figure S2e), while SEH1L showed a significant negative correlation with this parameter (r = 0.48, *p* < 0.05) (Supplementary Figure S2f).

## 4. Discussion

In this study we revealed a quantitative shared impact of cannabis use and SCZ. These results are consistent with recent GWAS studies showing a significant common genetic risk of SCZ and cannabis use [29]. Interestingly, when assessing the exclusively deregulated proteins, we observed a functional selectivity among the different groups. Thus, the most significantly deregulated pathway in C/c was the immune system cascade, while in SCZ/c metabolism of RNA, cell responses to external stimuli and metabolism of proteins showed strong and significant alterations. In SCZ/nc, smaller but significant deregulations were observed in cell cycle, DNA replication and cell responses to external stimuli cascades. These findings indicate that despite the common proteomic changes induced by cannabis use and SCZ, there might be specific changes prompted in the proteome depending on whether these two factors are present together or separately. In fact, when we subtracted the effect of cannabis comparing both groups of SCZ patients with C/c, we found a more similar profile between SCZ/nc and C/c, with small deregulations in protein localization and signal transduction, than in SCZ/c and C/c, which showed larger changes only in metabolism of RNA. Moreover, when the exclusively deregulated proteins were compared in both groups of SCZ patients, differences were obtained for metabolism of RNA and cellular responses to external stimuli. Again, these comparisons revealed functional differences in the proteomic alterations induced by the presence of SCZ depending on whether patients use cannabis or not.

Therefore, to identify specific proteins that could be proteomic markers of SCZ with or without the concomitant use of cannabis, we assessed separately the commonly deregulated proteins in SCZ/nc and in SCZ/c with respect to all the other groups. In SCZ/nc, we found three protein markers: CDK5, LSM2 and YARS2. These proteins were significantly downregulated in SCZ/nc, but not in SCZ/c. CDK5 is a cyclin-dependent kinase that controls the development of the central nervous system [34]. In the mature brain, it is associated with cognitive processes [35], and in vitro, it modulates oxidative stress responses [36]. According to the REACTOME database, it participates in developmental biology, which functionally differentiates SCZ/nc from SCZ/c. The observed reduction in CDK5 expression is consistent with previous studies showing a significant decline in CDK5 expression in postmortem brain samples of antipsychotic-treated SCZ patients, but not in drug-naïve individuals [37]. Markedly, our data indicate that cannabis use may counteract the effects of SCZ on CDK5. Further studies may shed light on whether cannabis use also opposes antipsychotic efficiency through this mechanism. Secondly, the downregulation of LSM2, which encodes for a key protein of the spliceosome [38], is in agreement with previously reported global changes in alternative splicing in SCZ patients [39]. LSM2 participates in metabolism of RNA, which differentiated SCZ/nc from SCZ/c, and presented a positive correlation with GAF and a negative correlation with NSS, indicating that it may be relevant for the clinical alterations observed in SCZ. Thirdly, we show a decrease in YARS2, a tyrosyl-tRNA synthetase located in the mitochondria, involved in metabolism of proteins, which differentiated SCZ/nc from SCZ/c. Its downregulation may lead to respiratory chain dysfunctions causing mitochondrial oxidative stress [40], which has been observed in postmortem brain samples from SCZ patients [41,42]. Moreover, lower levels of YARS2 could be correlated with worse attention performance, consistent with previous studies reporting that an oxidative imbalance increases negative symptoms’ severity in SCZ patients [43]. Once more, cannabis use seems to counteract the molecular changes in YARS2 in ON cells of SCZ/c, hypothetically via CB_1_ receptors expressed in the mitochondrial membranes [44], which have been proven to modulate respiration in this organelle [45].

In SCZ/c, we identified expression level changes in seven proteins: CDK5RAP3, HDHD5, MYO1B, MTREX, RPS20, SEH1L, and ZNF326. CDK5RAP3, a regulator of CDK5, was significantly upregulated in SCZ/c with respect to all other groups. It is genetically associated with SCZ [46], and participates in cell cycle. It modulates hippocampal aging because its transcriptional upregulation lowers neurogenesis [47]. Higher levels of this protein were associated with less GAF and more NSS in the entire population. MYO1B, SEH1L and RPS20 were also deregulated, specifically in SCZ/c, and were associated with clinical outcomes (GAF and NSS). They are involved in the metabolism of proteins cascade, which includes the AKT-mTOR pathway that is thought to play a role in the interaction between SCZ and cannabinoids exposure in humans [48] and in mice models [24]. In fact, MYO1B, SEH1L and RPS20 directly or indirectly interact with AKT-mTOR [49,50,51]. Interestingly, we found a negative correlation between MYO1B and plasmatic concentrations of THC-COOH in SCZ/c and C/c. THC-COOH is the last molecule arising from ∆^9^-tetrahidrocannabinol degradation [52], which might point to the role of this unconventional myosin in cannabis metabolism. Finally, MTREX and ZNF326, which are involved in the metabolism of RNA cascade, were found to be deregulated in SCZ/c as compared to the rest of the groups. In fact, metabolism of RNA was consistently deregulated in SCZ/c in every functional comparison. Although prior studies have found deregulations in ribosomal and RNA-related proteins in human samples from SCZ patients [53,54], our data revealed functional differences in metabolism of RNA exclusively linked to the coexistence of cannabis consumption and SCZ. According to REACTOME, MTREX and ZNF326 are involved in the same step of pre-mRNA processing prior to protein translation. However, we found that MTREX was significantly downregulated in SCZ/nc and SCZ/c, while ZNF326 was upregulated in SCZ/c, but both of these alterations correlated with worse cognitive performance and more clinical signs of SCZ. Thus, a lower peak intensity of MTREX was associated with worse spatial attention, verbal fluency, and GAF scores, but with more NSS. On the other hand, higher peak intensity levels of ZNF326 were related with worse attention, working memory, emotional recognition, and clinical signs. Moreover, cannabis use duration positively correlated with ZNF326 expression. These data indicate that either a decrease in MTREX or an increase in ZNF326 expression may have a negative effect on RNA metabolism, which could impact neurocognitive functioning.

The results of our study need to be interpreted considering its strengths and limitations. The main strength of our data relies on the translational power of ON cell models to investigate biomarkers of neuropsychiatric disorders, such as SCZ. In addition, the evaluation of cannabis’ effects on separate cohorts of SCZ patients is unique, since there is an absence of studies comparing these populations. The main limitation of our study was the small number of subjects per group, thus, additional follow-up studies on a larger scale, including longitudinal and epidemiological studies, will be needed to further corroborate these findings.

In summary, we revealed a quantitative shared effect of SCZ and cannabis use in the proteomic profile of ON cells, consistent with the genetic similarities previously described [29]. We found that cannabis use in controls has a significant impact on the immune system, while it alters metabolism of RNA and metabolism of proteins in SCZ patients. Additionally, SCZ/nc show small but significant differences in cell cycle, DNA replication, proteins localization and signal transduction. In this group, we identified significant changes in two functionally relevant proteins (YARS2 and LSM2) associated to attentional processes, GAF and NSS scores. On the other hand, in ON cells from SCZ/c we found significant changes in six proteins relevant to clinical scores (MYO1B, MTREX, ZNF326, RPS20, CDK5RAP3, SEH1L) and two for cannabis use (MYO1B, ZNF326). Moreover, in SCZ/c, we identified consistent deregulation in metabolism of RNA in every comparison, and expression levels of MTREX and ZNF326, which take part in this pathway, correlated with several aspects of cognitive performance.

## Figures and Tables

**Table 1:** Demographic characteristics of the different groups included in the study.

	Control Subjects (C/nc)	Cannabis Users (C/c)	Schizophrenia Non-Cannabis (SCZ/nc)	Schizophrenia Cannabis (SCZ/c)
Age (years)	31.4 ± 5.5	29.2 ± 5.6	37 ± 10.1	41.4 ± 6.2
Gender (M-F)	3/2	4/1	2/3	5/0
**Tobacco use**				
Users—n (%)	5 (100%)	4 (80%)	4 (80%)	5 (100%)
Units per week ^µ^	53.8 ± 50.2	36.4 ± 40.6	91.2 ± 91.0	147 ± 62.6
Use duration (years) ^µ^	11.4 ± 4.9	10.4 ± 10.0	5.8 ± 6.8	24.2 ± 9.6 *^,+,#^
**Cannabis use**				
Age first use (years) ^µ^	-	15.4 ± 2.5	-	17.2 ± 3
Units per week ^µ^	-	16.2 ± 10.2	-	19.4 ± 14.6
Use duration (years) ^µ^	-	12.2 ± 7.2	-	17.6 ± 11.5
**Antipsychotic treatment**				
Clozapine/Olanzapine	-	-	1/5	3/5
Aripiprazole	-	-	2/5	1/5
Paliperidone	-	-	1/5	1/5
Risperidone	-	-	1/5	-

Data are shown as mean ± SD. * *p* < 0.05 vs. C/nc; +*p* < 0.05 vs. C/c; ^#^ *p* < 0.05 vs. SCZ/nc. (µ) Indicates continuous non-normally distributed variables.

**Table 2:** Neuropsychological and clinical data of the different groups.

	Control Subjects (C/nc)	Cannabis Users (C/c)	Schizophrenia Non-Cannabis (SCZ/nc)	Schizophrenia Cannabis (SCZ/c)
Direct series score	8.8 ± 0.4	8.2 ± 1.8	7.4 ± 1.5	8.8 ± 2.2
Inverse series score ^µ^	6.3 ± 1.3	8.2 ± 5.5	4.4 ± 1.5	8.8 ± 2.1
Verbal fluency score	26 ± 1.2	22.8 ± 4.5	18.8 ± 6.9	17.2 ± 5.3 *^,+^
Direct spatial span	7.4 ± 0.6	5.6 ± 1.9	5.2 ± 1.5	5.6 ± 1.7
Inverse spatial span	7.6 ± 2.9	5.8 ± 1.5	5.6 ± 3	5.6 ± 1.7
Emotional recognition	65.9 ± 8.6	73.8 ± 7.4	58.2 ± 22.5	58.9 ± 10.2
Global assessment of functioning ^µ^	99 ± 5	96 ± 5.5	67 ± 8.4 ***^,+,++^	63 ± 2.7 ***^,+,++^
Neurological soft signs ^µ^	2.8 ± 1.1	5.2 ± 3.1	14.8 ± 8.6 **^,+^	13.2 ± 2.3 **^,+^

Data are shown as mean ± SD of the mean. * *p* < 0.05, ** *p* < 0.01, *** *p* < 0.001 versus C/nc; + *p* < 0.05; ++ *p* < 0.01 versus C/c. (µ) Indicates continuous non-normally distributed variables.

## Data Availability

In All MS raw data and search results files have been deposited into the ProteomeXchange Consortium (http://proteomecentral.proteomexchange.org) via the PRIDE partner repository with the dataset identifiers PXD020739 (Reviewer account details: Username: reviewer15955@ebi.ac.uk; Password: UDhwtjVV).

## Supplementary Materials

The following are available online at https://www.mdpi.com/2075-4426/11/3/160/s1, Supplementary Figure S1, Supplementary Figure S2, Supplementary Material.
