# Supplementary material for: Cannabis Use Induces Distinctive Proteomic Alterations in Olfactory Neuroepithelial Cells of Schizophrenia Patients
Source: J Pers Med. 2021 Feb 25;11(3):160. doi: 10.3390/jpm11030160 (PMC7996288; doi:10.3390/jpm11030160)
Supplement: Supplementary file 1 [file jpm-11-00160-s001.zip › Supp figure legends.docx]

**SUPPLEMENTARY FIGURE LEGENDS**

**Supplementary Figure 1.** Workflow diagram followed for the analyses of the proteomic data. Left panels show the deregulated proteins in each group when compared to HC/nc (top left panel); HC/c (central left panel) and SCZ/nc vs SCZ/c (bottom left panel). The exclusively deregulated proteins obtained in each analysis for SCZ/nc and SCZ/c were overlapped in order to obtain protein biomarkers of SCZ/nc and SCZ/c (central panel). Finally, the targeted proteins revealed in the analysis are represented in the right panels.

**Supplementary Figure 2.** Graphical representation of the spearman correlation analyses involving (a) CDK5RAP3 and GAF, (b) CDK5RAP3 and NSS, (c) MYO1B and GAF, (d) MYO1B and NSS, (e) RPS20 and GAF and (f) SEH1L and GAF.
