# Supplementary material for: Cannabis Use Induces Distinctive Proteomic Alterations in Olfactory Neuroepithelial Cells of Schizophrenia Patients
Source: J Pers Med. 2021 Feb 25;11(3):160. doi: 10.3390/jpm11030160 (PMC7996288; doi:10.3390/jpm11030160)
Supplement: Supplementary file 1 [file jpm-11-00160-s001.zip › Supp Information proteomic methodology.docx]

**SUPPLEMENTARY INFORMATION**

Cannabis Use Induces Distinctive Proteomic Alterations in Olfactory Neuroepithelial Cells

Of Schizophrenia Patients

Marta Barrera-Conde, MSc^1,3^, Karina Ausin, PhD^2^, Mercedes Lachén-Montes, PhD^2^, Joaquín Fernández-Irigoyen, PhD^2^, Liliana Galindo, PhD^5,6^, Aida Cuenca-Royo, PhD^1^, Cristina Fernández-Avilés^3^, Víctor Pérez, MD, PhD^6,7^, Rafael de la Torre, PhD^1,3,4^, Enrique Santamaría, PhD^2^ and Patricia Robledo, PhD^1,3*^

**METHODS FOR PROTEOMIC STUDIES**

The homogenates were spun down at 100.000 × g for 1 h at 15 °C and protein concentration was measured by Bradford assay (Bio-rad). Pool of aliquots of all samples in the sample sets were used as input for generating the SWATH-MS assay library. To increment the proteome coverage, in-solution and in-gel digestion workflows were applied. In the first one, protein material from each pooled sample was precipitated using methanol/chloroform extraction. Pellet was dissolved in 6 M urea, 100mM Tris, pH 7.8. Reduction was performed by addition of DTT to a final concentration of 10mM and incubation at 25ºC for 1 h. Subsequent alkylation by 30 mM iodoacetamide was performed for 1h in the dark. An additional reduction step was performed by 30mM DTT, allowing the reaction to stand at 25ºC for 1h. The mixture was diluted to 0.6M urea using MilliQ-water, and after trypsin addition (Promega) (enzyme:protein, 1:50 w/w), the sample was incubated at 37ºC for 16h. Digestion was quenched by acidification with acetic acid. The digestion mixture was dried in a SpeedVac, reconstituted with 40 ul of 5 mM ammonium bicarbonate (ABC) pH 9.8, and injected to an ÄKTA pure 25 system (GE Healthcare Life Sciences) with a high pH stable X-Terra RP18 column (C18; 2.1 mm × 150 mm; 3.5 μm) (Waters). Peptides were eluted with a mobile phase B (5mM ammonium formate in 90% ACN at pH 9.8) of 30-70% linear gradient over 45 min (A, 5mM ammonium formate in water at pH 9.8; B, 5mM ammonium formate in 90% ACN at pH 9.8). The UV signal was monitored at 215nm. Fractions were collected and evaporated under vacuum. Neighboring fractions with the low signal intensity were subsequently pooled to generate fractions with similar peptide content. For the second digestion protocol, protein extracts (20 ug) were diluted in Laemmli sample buffer and loaded into a 0.75 mm thick polyacrylamide gel with a 4% stacking gel casted over a 12.5% resolving gel. Total gel was stained with Coomassie Brilliant Blue and 12 equals slides from each pooled sample were excised from the gel and transferred into 1.5 mL Eppendorf tubes. Protein enzymatic cleavage was carried out with trypsin (Promega; 1:20, w/w) at 37 °C for 16 h as previously described^1^. Purification and concentration of peptides were performed using C18 Zip Tip Solid Phase Extraction (Millipore) and, when recovered from in-gel and in-solution digestion processing peptides were reconstituted into a final concentration of 0.5µg/µL of 2% ACN, 0.5% FA, 97.5% MilliQ-water prior to mass spectrometric analysis. MS/MS datasets for spectral library generation were acquired on a TripleTOF 5600+ mass spectrometer (Sciex) interfaced to an Eksigent nanoLC ultra 2D pump system (Sciex) fitted with a 75 μm ID column (Thermo Scientific 0.075 × 250mm, particle size 3 μm and pore size 100 Å). Prior to separation, the peptides were concentrated on a C18 precolumn (Thermo Scientific 0.1 × 50mm, particle size 5 μm and pore size 100 Å). Mobile phases were 100% water 0.1% formic acid (FA) (buffer A) and 100% Acetonitrile 0.1% FA (buffer B). Column gradient was developed in a gradient from 2% B to 40% B in 120 min. Column was equilibrated in 95% B for 10 min and 2% B for 10 min. During all process, precolumn was in line with column and flow maintained all along the gradient at 300 nl/min. Output of the separation column was directly coupled to nano-electrospray source.

**REFERENCES**

1 Shevchenko A, Tomas H, Havliš J, Olsen J V., Mann M. In-gel digestion for mass spectrometric characterization of proteins and proteomes. *Nat Protoc* 2007; **1**: 2856–2860.
