# Supplementary figures and images for: Cannabis Use Induces Distinctive Proteomic Alterations in Olfactory Neuroepithelial Cells of Schizophrenia Patients
Source: J Pers Med. 2021 Feb 25;11(3):160. doi: 10.3390/jpm11030160 (PMC7996288; doi:10.3390/jpm11030160)

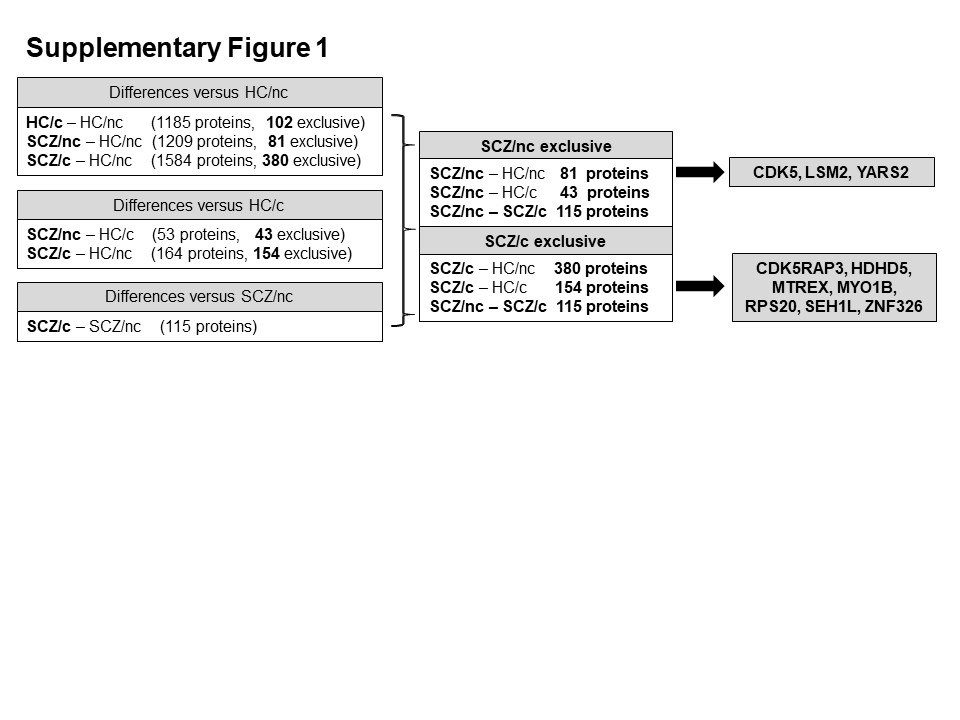

Supplement: Supplementary file 1 [file jpm-11-00160-s001.zip › Supp Fig 1.jpg]

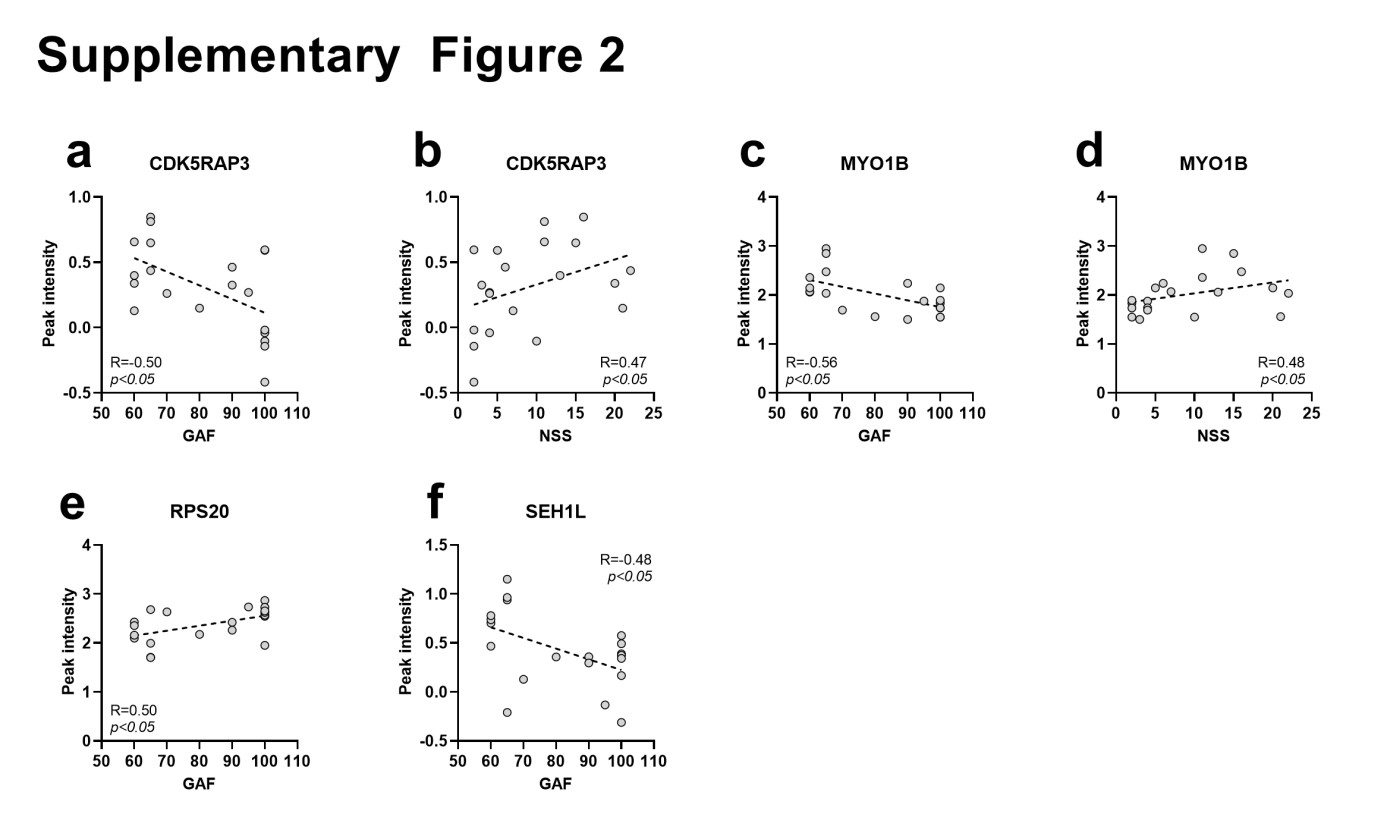

Supplement: Supplementary file 1 [file jpm-11-00160-s001.zip › Supp Fig 2.jpg]
